# Photocurable Polymer Coatings from Renewable Sources – Perspective Applications for Electronics, Surface Protection, and Advanced Systems

**DOI:** 10.1021/acsmaterialsau.6c00087

**Published:** 2026-07-15

**Authors:** Vojtěch Jašek, Radek Přikryl

**Affiliations:** Institute of Materials Chemistry, Faculty of Chemistry, 48274 Brno University of Technology, 61200 Brno, Czech Republic

**Keywords:** Photocuring, Bio-Based, Electronic, Protective Coatings, Advanced Coatings, Photopolymerization

## Abstract

The photocurable coating industry relies mostly on fossil-based, high-performing, and multifunctional polymerizable systems, often comprising acrylic, epoxy, polyurethane, and unsaturated structural components suitable for efficient photopolymerization, exhibiting stable chemical structures and sufficient reproducibility. This work offers a broad discussion of reactive systems derived and synthesized from renewable sources. The main aim is to introduce different structural precursors and employ various photopolymerization strategies, including radical and cationic polymerization and thiol–ene photocuring. Besides the reported inventions of biobased photocurable precursors, exemplified only by assisting matrices and the substitution of petroleum-derived photopolymerized materials, our work targeted and evaluated functional components from renewable sources and introduced their irreplaceable and unique purpose across commercial, material, and advanced applications incorporating photocurable polymer coatings. We divided the discussed precursors into utility-focused subsections and comprehensively evaluated their primary roles in the application fields.

## Graphical abstract

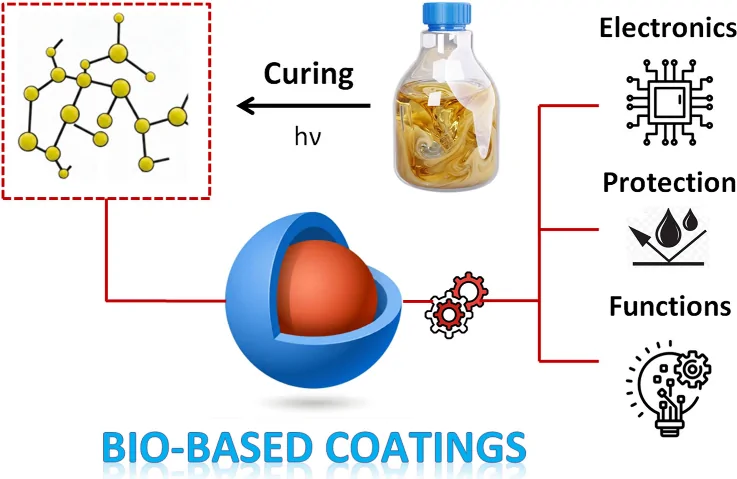

## 1 Introduction

Photocurable polymer coatings exemplify an important role across several application fields and material sectors. Their reactivity to UV or visible light initiates various polymerization mechanisms, leading to the formation of solid-phase layers, including free radical and cationic polymerization.^1–4^ Next to the polymerization mechanism, which differs in terms of reaction rate or the chemical composition of the photoinitiators used, numerous other physicochemical parameters are essential for coating precursors and must be taken into account. The rheological profile of the coating precursor or prepolymer used is one of the most crucial parameters, as it directly affects the final thickness of the forming layer, the efficiency of the coating process, and process control.^5,6^ Additionally, viscosity varies crucially with the actual temperature; therefore, precise temperature conditions must be set for both the photocurable precursor and the coated substrate.^7,8^ Generally, viscosity decreases with increasing temperature, affecting the coating process tremendously; however, the reactive systems tend to undergo spontaneous polymerization since they bear specific functional groups ensuring their photocurability.^9,10^ The precise monitoring of the overall temperature is important to avoid any undesired polymerization, often exhibiting exothermic character that accelerates the chemical reaction further.^11,12^ The eventual thickness of the engineered coating is primarily affected by the viscosity of the precursor and the coating process, and affects many of the following processing steps. The conversion of reactive functional groups into the eventual polymer structure depends on the overall penetration depth of the irradiation source, which is determined by the absorption of the employed photocurable system. When compounds with inappropriate chromophores are used, the photocuring process is severely compromised.^123^ Also, if short-chain polymerizable precursors are used, the efficiency of the photocuring (or the rate of photopolymerization) can be significantly higher due to the increased diffusivity of carbon chains. Once the polymer precursors turn into a hard resin, the molecular mobility of the remaining polymerizable groups is limited. When high-molecular-weight precursors are used, their incorporation into the 3D molecular polymer site of the photocured thermoset may require longer time and may lead to lower degrees of cure. Therefore, photoinitiated polymerization requires precise optimization to account for the mechanical properties of the formed resin.^13,14,122^ Besides the polymerization process, the thicker-coated layers naturally contain more cured material, which increases cost and the potential environmental impact in applications that could use less polymerized material.^15^

Adhesion to the coated substrate is one of the most essential process parameters. The optimal interface chemistry, determined by the intermolecular interactions between the coated precursor and the target substrate, dictates the final properties of the solid-phase polymer.^16–19^ The polar substrates, such as wood, paper, glass, or specific metals, exhibit high surface energy and require polar-coated media for optimal performance.^20–22^ Typically, the functional groups promoting dipole–dipole (Keesom), dipole–induced dipole (Debye) interactions, and hydrogen bonding work the best with such substrates (see Figure 1). Hydroxyl (−OH), amine (−NH_2_), amide (−CO–NH−), ester (−COOR′), and carbamate (−NH–C­(=O)–O−) functional groups belong among the strong polar functional groups.^23–26^ On the other hand, the nonpolar adherents form primarily induced dipole–induced dipole interactions (London/dispersion), exhibit lower surface energies (typically polypropylene (PP), polyethylene (PE), poly­(methyl methacrylate) (PMMA), or polytetrafluoroethylene (PTFE), and prefer nonpolar liquid precursors for the optimal coating process, including fluorinated acrylates, long-chain esters (triacylglycerides, fatty acid monoesters), and nonpolar terminated polyimides.^27–30^ The optimal adhesion ensures efficient wetting of the adherent’s surface, filling every material defect and forming a compact solid–liquid interface. Once the precursor is photocured, the optimal coating remains strongly attached to the target substrate.^31,32^ Polymerization shrinkage is a potential issue for engineered photocured coatings, leading to coating failures and surface defects.^33,34^ The percentage of eventual shrinkage differs with the applied precursor (acrylate systems reach 2–14% of shrinkage: epoxy, polyurethane, and thiol–ene systems generally reach lower values up to 7–8%).^35–37^ Nevertheless, when the coated precursor is optimally designed for the target adherent, polymerization shrinkage becomes much less of a concern. In addition to the optimal chemical engineering of the applied liquid precursor, several solid-phase additives can be introduced into the photocurable system to address potential issues by improving the material interface.^38–40^

**Figure 1:**
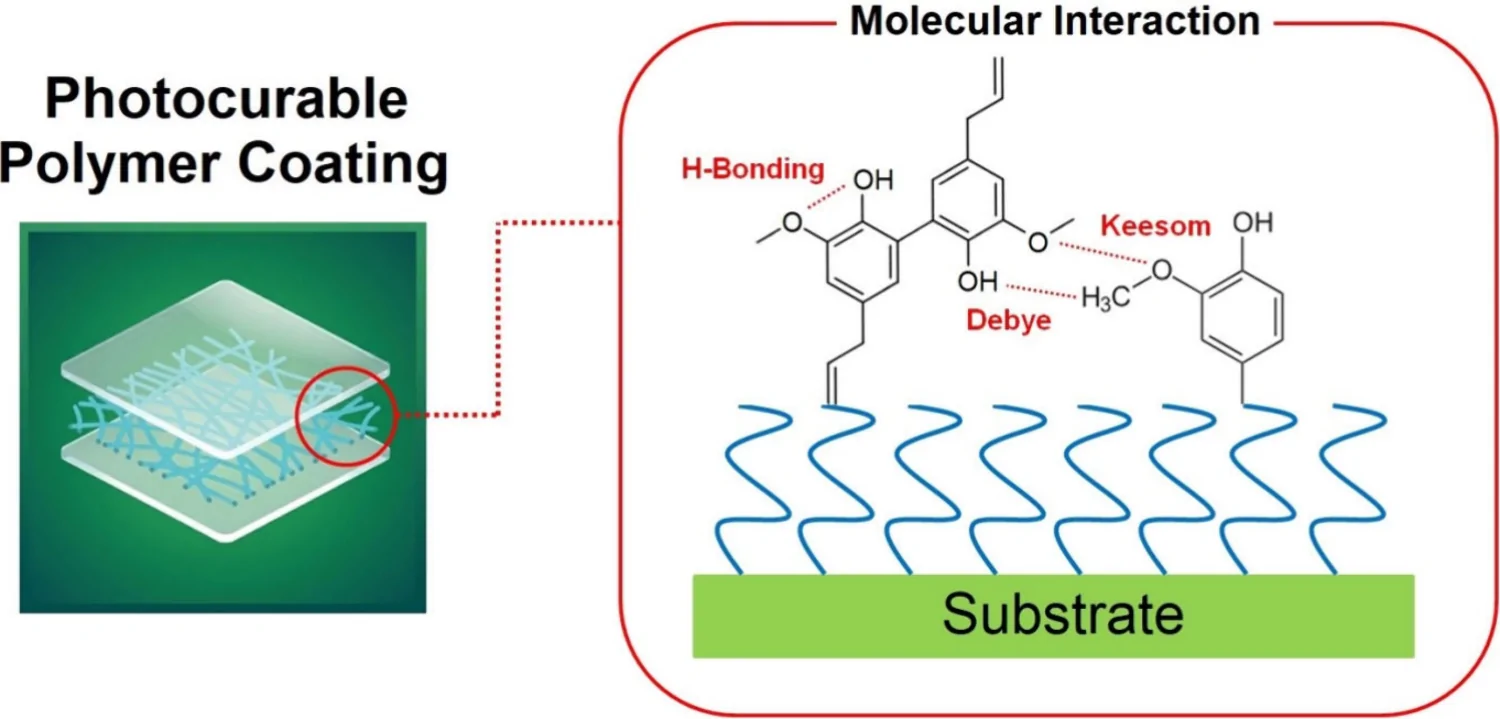
Illustrative intermolecular interactions forming among molecules of the coated polymer structures and the target substrate.

Acrylate and methacrylate systems are the most widely used derivatives for photocurable coating materials due to their well-known polymerization mechanisms, efficient photoinitiation, and fast curing rates.^41–44^ Acrylic derivatives typically exhibit higher polymerization rates but worse oxygen inhibition compared to the methacrylic systems.^45,46^ Additionally, the acrylic derivatives pose greater health hazards and environmental impacts due to their higher water solubility and volatility compared to the methacrylate systems.^47–49^ Regarding the chemical structures of the starting compounds, several different systems and derivatives are synthesized, each with numerous functional groups that affect the final viscosity, adhesion, and properties. Polyester-acrylic/methacrylic systems are the most common, straightforward, and widely investigated macromolecular structures used in coating science.^50–52^ The synthesis typically involves simple Fischer esterification or transesterification.^53,54^ Such structures can contain unmodified hydroxyl groups, amines, or long carbon chains, which determine the overall surface tension in the liquid state, the surface energy in the polymerized form, and the final adhesion and coating performance with the target adherent.^55,56^ Also, the unmodified functional groups in acrylic/methacrylic systems significantly affect photopolymerization activity.^57,58^

In addition to the polyester acrylates/methacrylates, several researchers have introduced and investigated many hybrid polyurethane esters. Polyurethanes contain carbamate linkages, exhibiting tough properties, varying strength levels, and superior adhesion to various substrates.^59,60^ The fusion of the conveniently photopolymerizable acrylates/methacrylates together with the intermolecular interactions of polyurethane compounds ensures a high performance of the produced thin layers.^59,60^ Epoxy systems and their hybrids with acrylic/methacrylic functional groups represent the major group of photopolymerizable precursors for the coating industry.^61–63^ The pure epoxy precursors are typically polymerized via cationic photopolymerization, which involves functionally distinct photoinitiators and generally yields lower curing rates than the acrylic/methacrylic systems.^64,65^ Epoxy-acrylic hybrids offer a unique opportunity for frontal polymerization systems that involve two separate processes. First, the radically polymerizable acrylates/methacrylates form a thermoset structure, bearing unpolymerized epoxy functional groups within the carbon backbone. Second, the free epoxides are initiated in cationic polymerization, enhancing the eventual dual-cured structure. Frontally polymerized systems are among the most vigorously studied multicomponent systems and could potentially serve specialized coating purposes.^66,67^

This work presents several suggested and experimentally investigated photocurable precursors for coating applications derived from natural biosources, representing a functional and sustainable alternative to the currently used systems. The specific applications represent the main discussion sections, as many acrylic, methacrylic, epoxy, polyurethane, and various hybrid systems have been previously introduced and reported. However, most of these precursors and their fabricated coatings have not been applied to a particular utility. This work evaluated the properties, sustainability, and coating performance in electronics and optics, surface protection and industrial decorative finishes, and special advanced applications (illustrated in Figure 2). We incorporate all structural types of engineering coatings, primarily using biobased starting compounds or systems specifically modified for the target application.

**Figure 2:**
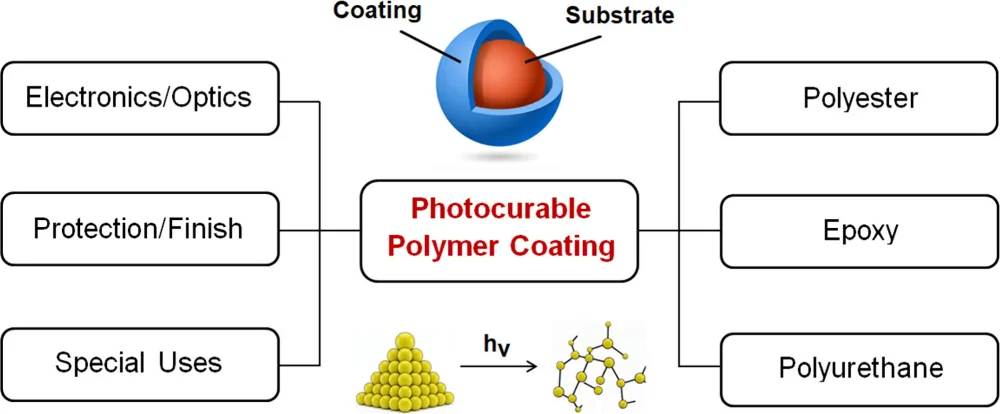
This work presents a schematic illustration of target applications and the chemical types of photocurable polymer coatings.

## 2 Electronics and Optics

Electronics and optics require several functions from the applied photopolymerizable polymer coatings, namely an outer protection from specific environments (typically moisture, oxidative atmospheres, solid particle dusts, or corrosive compounds and systems),^68^ insulating function (generally to reduce electromagnetic interference),^69^ specific encapsulation (providing selectively penetrable layers, protecting the substrate from specific factors),^70^ or specific surface modification (to response to particular stimuli transferring through the electronic device).^71^ Particular experimental investigations covering polymer coating for electronic and high-end applications, such as binders for Li-Ion batteries,^120^ nanocomposites,^120^ or for applications requiring resistance to high-cycle creep fatigue.^121^ The biobased photocurable systems were designed and experimentally tested in the published literature.^77–79^ Generally, various colorings arising from renewable, natural starting compounds may complicate the final properties of the coating; biobased alternatives to fossil-based systems often exhibit lower reactivity, and nonoptimal rheological profiles can also complicate the fabrication of thin layers.^72–74^ On the other hand, lower VOC generation is the main advantage of photocurable coatings over solvent-assisted thermoplastic systems.^75^ Moreover, when renewable starting compounds are used, the eventual polymer coating contains more bioderived carbon and weight content. These factors significantly contribute to overall sustainability in the polymer industry, which is currently attracting widespread attention.^76^

Various renewable templates served as starting materials for many photocurable polymer coatings used in electronics. Customized triboelectric nanogenerators (TENGs) are currently focused on innovative solutions that emphasize the sustainability of the reactive precursors used. The cutting-edge research reported high-resolution printability for surface usages in TENGs, incorporating starting compounds, such as palm oil (PO) and phytic acid (PA).^77^ The researchers reported the synthesis of a methacrylate PO derivative (MPOEA) and a PA-based methacrylate monomer (GPA) developed through molecular engineering and specific structural modification of the selected renewable templates. The proposed photocurable system has sufficiently low viscosity for optimal coating applications (<42 mPa·s) and high postpolymerization conversion (96.8%). The quantified biobased content exceeded 60%, and the photocuring achieves a rapid curing rate of less than 30 s. The prepared polymer system also exhibited excellent electrical performance, peak power density, and exceptional operational stability. The authors indisputably demonstrated that photocurable systems with high natural content represent promising alternatives to the currently employed petroleum-based reactive systems.

Besides the naturally produced complex acids and modified vegetable triacylglycerides, other researchers combined hindered urea bonds (HUB) with radically polymerizable biobased precursors fabricated from a cellulose-incorporating prepolymer.^78^ Additionally, the authors also employed oil-based monomers to represent “soft phases” in the engineered thermosets, while the cellulose segments exemplified “hard phases” (see Figure 3). The eventual biobased photocurable materials exhibited remarkable properties, including high toughness (2.89 MJ·m^–3^), excellent malleability and reprocessability, and a 94.7% recovery efficiency. The authors used their engineered, multicomponent photocurable system, in combination with suspended silver particles within the polymerizable continuum, to produce a thermoset-silver composite electronic coating with an exceptional Joule-heating effect for an anti-icing or deicing device. Moreover, their proposed polymer-based electronic systems can be used as capacitive sensors with reliable, sensitive performance for real-time monitoring of environmental humidity, human respiration, and human motion. The combination of the optimal rheological profile, the macro-cross-linking character of the “soft” and “hard” segments of the synthesized biobased thermoset precursor, and the exceptional physical-chemical properties of the cured resin offers a promising design for new-generation multifunctional electronic devices.

**Figure 3:**
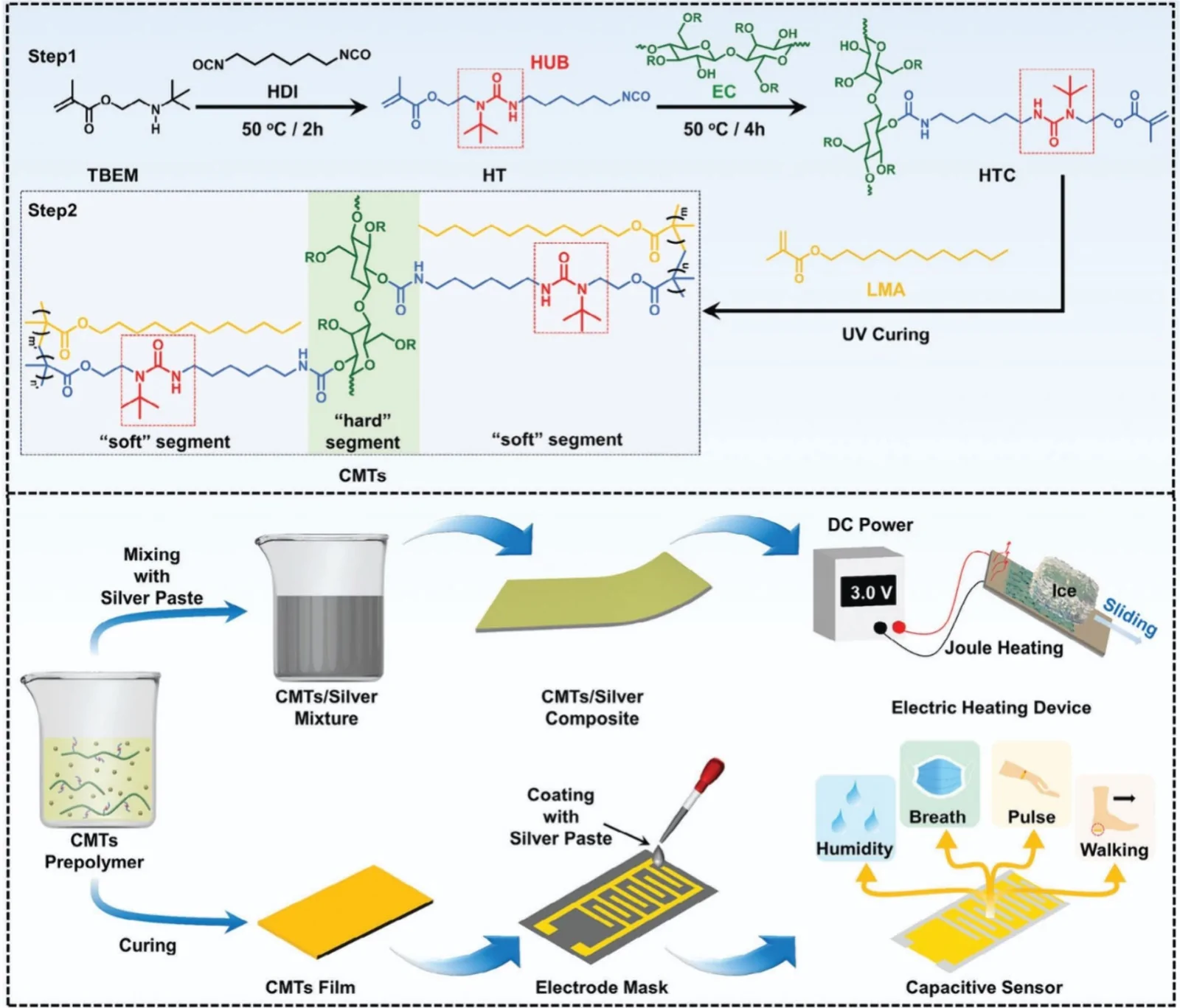
Proposed and investigated photocurable multicomponent biobased precursors were fabricated from “soft” segments comprising lauryl methacrylate (LMA) and hindered urea bonds (HUB), and “hard” segments based on ethyl cellulose (EC). The reactive system also comprised 2-(*tert*-butylamino)­ethyl methacrylate (TBEM) and hexamethylene diisocyanate (HDI). The target applications of the synthesized cellulose-based malleable biothermoset (CMT) include capacitive sensors and a CMT-silver composite for Joule heating.^78^ Adapted with permission from ref 78. Copyright 2023 Wiley-VCH GmbH.

Besides the previously discussed polyester^77^ and amide-involving structures,^78^ other synthetic approaches, including epoxides and thiol–ene click reactions, have been reported in the literature, producing biobased electronic packaging coatings (illustrated in Figure 4).^79^ The authors investigated the multistep modification of two selective lignin monomer derivatives, vanillyl alcohol (VA) and eugenol (EU). Both of these biobased compounds contain numerous functional groups, such as hydroxyl (−OH), alkoxy (R–OR′), and methylidene (=CH_2_). Eugenol, containing an unsaturated double bond, is an optimal starting compound for thiol–ene click modification by sulfur-containing systems. The investigators specifically used 2-hydroxy-2-methylpropiophenone (HMPP) to introduce an additional free hydroxyl group onto the eugenol carbon backbone. The functionalized EU structure was eventually modified into difunctional eugenol-derived epoxy (DGEEM). Vanillyl alcohol contains two modifiable hydroxyl groups, which were directly functionalized with epoxy using epichlorohydrin (ECH). The two produced diepoxy lignin derivatives were eventually acrylated by acrylic acid, following the “ring opening” nucleophilic substitution mechanism. The authors obtained biobased photosensitive intermediates with polymerizable acrylic groups, which were finally modified to form a biobased photocurable resin using tetrahydrophthalic anhydride (THPA). All synthesized reactive products were structurally characterized by cross-analysis using FT-IR and NMR. The achieved properties of the photocured thermoset included high tensile strength (28.89 ± 4.39 MPa), excellent thermal stability and heat resistance, a high glass transition temperature (*T*_g_ = 103.9 °C), optimal toughness (1.40 MJ·m^–3^), and a stable electrical breakdown strength of 222.87 kV·mm^–1^. The produced polymer precursors showed exceptional potential for sustainable, renewable electronic devices.

**Figure 4:**
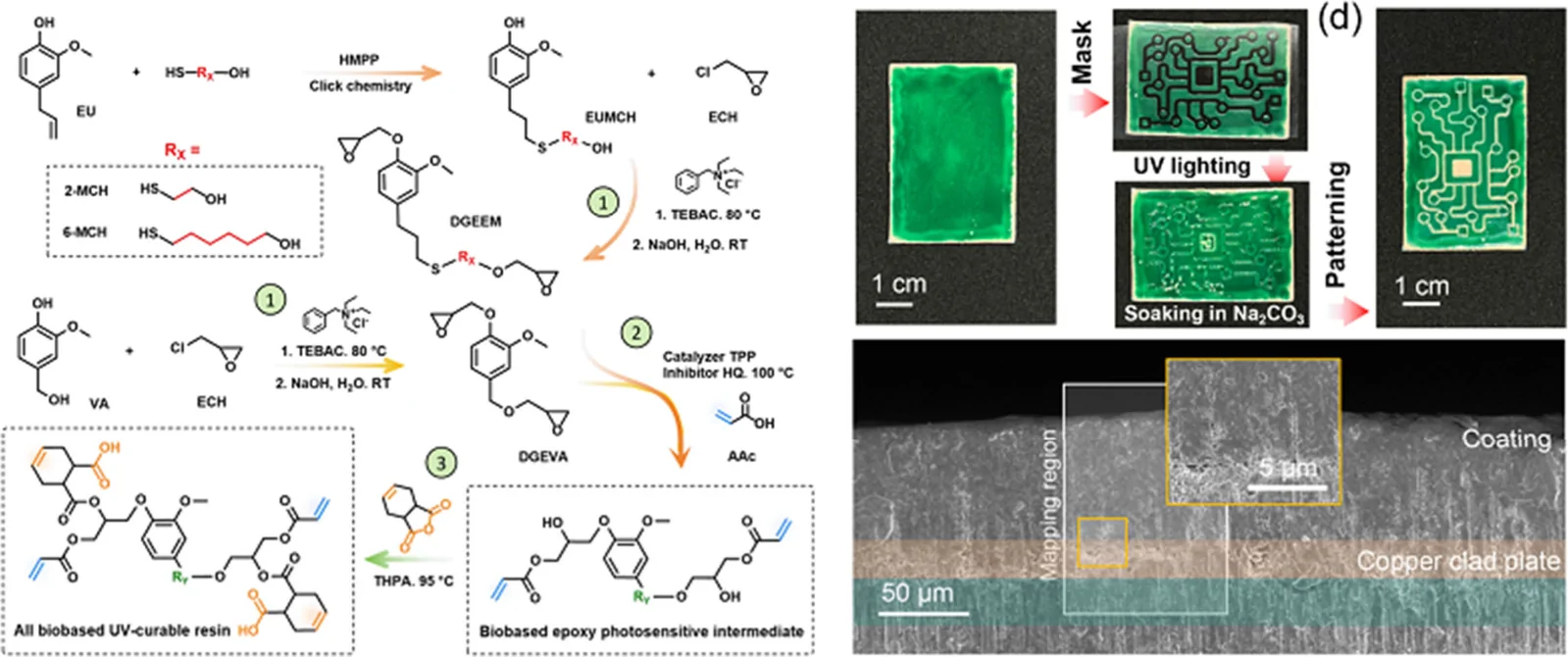
Multistep synthesis of lignin-based photocurable coatings produced from functionalized eugenol (EU) and vanillyl alcohol (VA), applicable in biobased electronic packaging coatings. The curable monomers were prepared from starting compounds by click chemistry, nucleophilic substitution incorporating epichlorohydrin, and the ring-opening modification of glycidyl derivatives with acrylic acid, followed by anhydride modification (nucleophilic substitution). The produced reactive precursors are photocured via radical polymerization.^79^ Adapted with permission from ref 79. Copyright 2024 American Chemical Society.

Electronic and optical products require several essential parameters to ensure proper function while avoiding undesired side effects. This perspective provides specific explored applications and investigated factors. The most significant aspects for this utility are summarized below:

- Polymer coatings usually serve as material carriers of the functional component in the final products. Therefore, their maximal optical clarity and minimal potential interferences are a priority for the eventual product.
- The combination of durability and high deformation resistivity is required due to the mainly protective and carrier character of polymer coatings.
- The applied polymer coatings should exhibit none or minimal electrochemical behavior to avoid negative interference with the final system.
- When biobased precursors and entering sources are considered, a minimal number of unnecessary chromophores is preferred to minimize limiting factors that negatively affect the final function.

## 3 Protection and Finishes

The modification of planar surfaces of various adherents, comprising materials with different properties, is essential in many applications. Photocurable precursors are often used as protective, decorative, finishing, or functional layers, serving as an in-contact surface with the surrounding environment that could damage or alter the encapsulated substrate.^80–83^ Different coated adherents exhibit specific characteristics and chemical compositions, thereby determining the most hazardous environments and substances for the encapsulated materials. Highly polar and organic systems, such as wood and paper, are typically fragile in wet conditions.^84,85^ Also, wood is composed of cellulose, lignocellulose, and lignin, of which the aromatic lignin content is highly affected by UV irradiation.^86–88^ The phenolic derivatives comprise several chromophores that absorb UV and even visible photons, resulting in irradiation aging.^87,88^ Eventually, wood materials are often used in interior furniture and decorative objects that require surface protection against mechanical damage.^89^ Several photocurable coatings exhibiting hydrophobicity, UV-absorption, and sufficient mechanical properties are applied in wood and paper substrates.^97–103^ Moisture-initiated corrosive processes damage several metal materials and products. Resistant photocurable coatings effectively serve as an anticorrosion layer, protecting metal surfaces from oxidation and corrosion.^90,91^ In addition to the mentioned uses, several polymer-derived coatings also provide self-healing character to the treated surface, perfectly serving decorative and finishing purposes.^92,93^ Finally, specific advanced applications involving metal, glass, or organic functional components, such as electrodes, probes, electrical circuits, or windows, may employ molecularly engineered photocurable coatings tailored to the particular application.^94–96^

Wood substrates, using biobased photocurable polymer coatings rather than fossil-derived coatings, naturally represent an optimal application target for such sustainable coatings due to their similar organic material structure and polar surfaces with high surface energy.^104^ Due to the similar chemical structure, optimal adhesion can be conveniently accomplished. Typical application fields for wood products protected by photocurable biobased coating are floorings and parquets (usually highly damaged by abrasion and also the UV absorption),^105^ exterior wood objects and surfaces (exhibited moisture and wet environment along with the fouling sources and irradiation exposure),^106^ interior furniture and design objects (mentioned earlier, representing visual design and suffering from UV-irradiation and abrasion),^107^ and specific wood-involving engineered products and composites (typically wood-plastic composites, plywood, or fiberboard).^108^ Many experimental investigations focused on the specific and universally engineered renewable photocurable polymer precursors. We listed the most up-to-date researchers and their applications in Table 1.

**Table 1:** Published Investigations Employing Bio-Based Starting Compounds in the Molecular Engineering of the Photocurable Wood Coatings

renewable compounds	bio-based content	type of the photocurable system	application	ref
rapeseed, linseed, and grapeseed oils, glycerol	15–75%	acrylates	high gloss wood coating	^97^
soybean oil, pentaerythritol	35–75%	acrylate and polyurethane	high gloss wood coating	^98^
capsanthin, soybean oil, eugenol	50–75%	thio-epoxy and thiol–ene	hydrophobic, antifungal wood coating	^99^
castor oil	48–72%	methacrylate	stain-resistant wood coating	^100^
cellulose filler	5–10%	methacrylate	hydrophobic, abrasion-resistant wood coating	^101^
tetrahydrofurfuryl, glycerol, soybean oil	7–40%	acrylate	abrasive-resistant wood coating	^102^
adipic acid, castor oil	57–79%	methacrylate, acrylate	stain-resistant wood coating	^103^

In addition to protective, abrasion- and stain-resistant, and decorative coatings for wood applications, biobased reactive compounds were successfully employed as anticorrosion layers for various metal-containing products and objects. Researchers prepared a photocurable coating precursor from epoxidized soybean oil and lignin to investigate lignin’s anticorrosive effect on their polymeric barriers.^109^ In their study, a triarylsulfonium hexafluorophosphate salt dissolved in propylene carbonate was used as a photoinitiator in coating formulations. Three lignin weight contents were incorporated: 0, 1, and 3 wt %. The authors performed cationic polymerization of epoxidized biobased precursors and investigated rheological characterization, cross-linking activity via photo-DSC, final epoxy conversion via FT-IR, thermal, surface, and anticorrosion performance of the prepared coatings. At the achieved epoxy-group conversion (about 75%), the investigators confirmed increasing coating hardness and adhesion to the steel substrate, improved hydrophobicity, an expressed antibacterial character, and a considerable anticorrosion performance for up to 2 weeks of immersion in the aggressive electrolyte with the 3 wt % lignin coating. Although anticorrosive properties were not investigated, another group of researchers employed biobased photocurable coatings on aluminum alloy surfaces to assess their functional performance.^110^ The main aim of the work was the application of acrylated, specifically methacrylated, vegetable oils (the methacrylate group was localized in a phosphorus-based compound) to iron–silicon-aluminum alloys to provide abrasive protection. Also, thermal stability was investigated, highlighting the positive aspect of the highly polar phosphorus group in the structure. The authors successfully prepared photocured polymer coatings with enhanced thermal stability, tensile properties, hardness, and hydrophobicity, which were mainly affected by the degree of cross-linking.

In addition to protecting highly polar and hydrophilic wood and organic substrates, and corrosion-susceptible metal objects and products, thermoplastic materials requiring specific surface treatments have also been reported in the published literature. The reported work focused on the application of photopolymerizable coatings comprising biobased acrylate compounds for the mechanical protection of polycarbonate (PC) substrates.^111^ The authors employed numerous industrially available low-molecular-weight photocurable monomers as alternatives to fossil-based, commercial precursors. Particularly, the following reactive multifunctional precursors with varying biobased content were investigated: dipentaerythritol penta-acrylate and dipentaerythritol hexaacrylate mixture (DiPEPHA, 15% biobased content), pentaerythritol tetraacrylate (PETA, 10% biobased content, tetra-functionality), 1,10-decanediol diacrylate (DDA, 60% biobased content, difunctionality), tetrahydrofurfuryl acrylate (THFA, 60% biobased content, monofunctionality), lauryl acrylate (LA, 80% biobased content, monofunctionality), isobornyl acrylate (IBOA, 75% biobased content, monofunctionality). The authors provided a comparison study, using tricyclodecane dimethanol diacrylate (TCDDA) as a fossil-based reference. The authors found that a decrease in cross-linking density significantly affected the prepared coatings, shifting the mechanical relaxation of the produced thin layers to higher temperatures for rigid IBOA and to lower temperatures for flexible THFA and LA. As summarized, 5 wt % loading is the optimal concentration of the employed monofunctional IBOA in the photocured coating.

The simple, straight photocurable precursors bearing specific reactive functional groups, such as acrylic, methacrylic, or epoxy, for thiol–ene reactions can be engineered to be more complex, employing multiple curing strategies beyond photocuring. Ding et al. published an investigation into biobased reactive systems capable of a dual-curing approach for step-by-step, continuous coating production.^112^ The authors used natural or (potentially) biobased components in their proposed photocurable, reactive systems, namely castor oil, tetrahydrofuran acrylate (THFA), polytetramethylene ether glycol (PTMEG), isophorone diisocyanate (IPDI), and hydroxyethyl methacrylate (HEMA). The molecular engineering of their suggested dual-curable systems comprises the polyaddition of castor oil with IPDI, forming CO-NCO (castor oil structure with excess −NCO functional groups), followed by a second addition modification with HEMA to the carbon backbone. The functionalized CO-NCO with unequimolar HEMA produced a reactive structure bearing methacrylate functional groups for the photocuring, and the excess isocyanate functional groups for the moisture curing. While photopolymerization requires a suitable photoinitiator and an irradiation source with an appropriate wavelength and exposure power, the moisture-initiated reaction (hydrolysis) requires water molecules, producing primary amines and carbon dioxide (see Figure 5). Using such a dual-curing strategy, the investigators produced oxygen-resistant polymer coatings with a stable chemical structure, a high glass transition temperature, and exceptional heat resistance. The coperformed mechanical tests and surface property investigations revealed improved final properties after dual curing compared to simple photocuring. The authors declare that their proposed complex systems are optimal for UV-curable polymerization under inadequate curing conditions.

**Figure 5:**
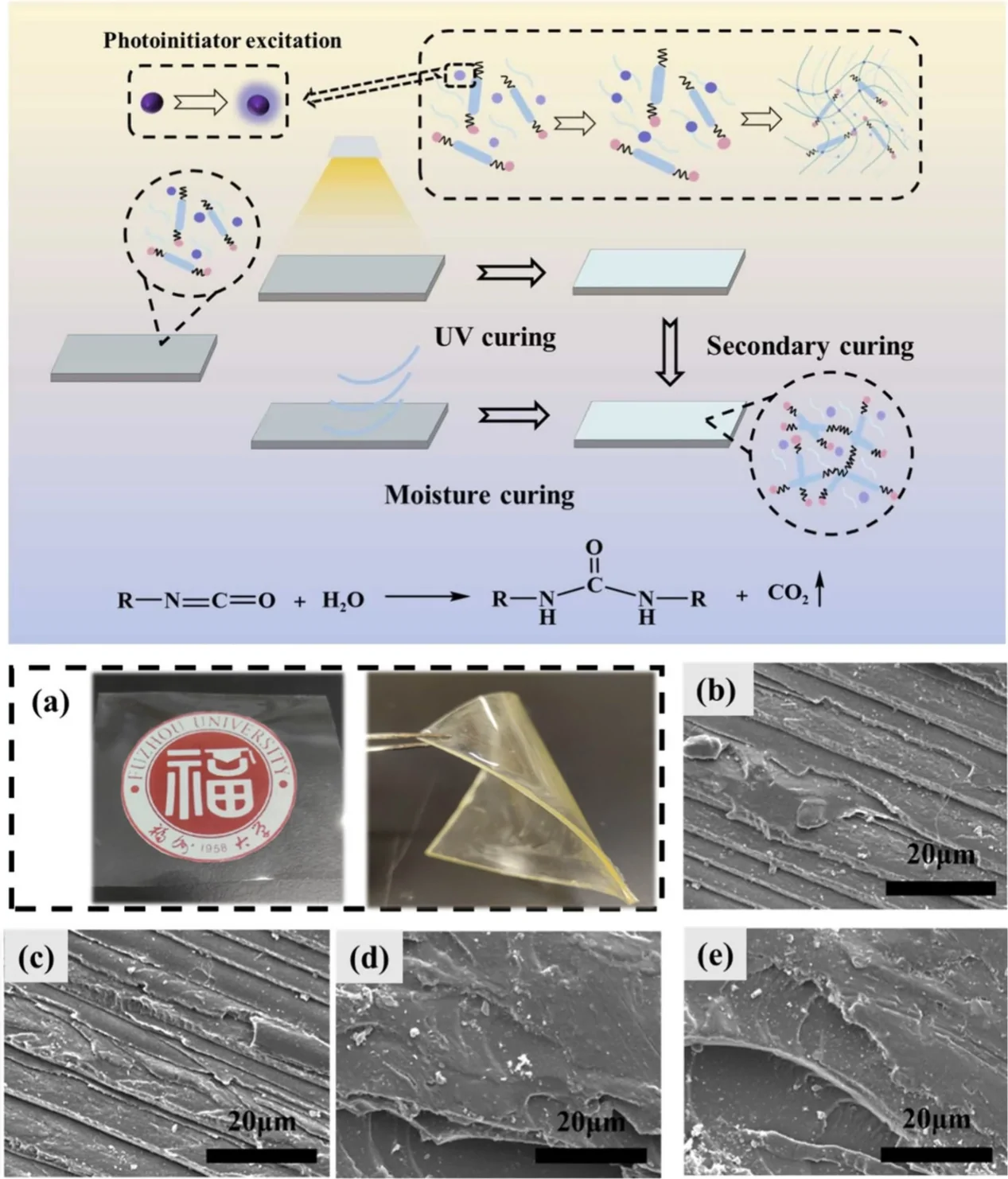
Proposed dual-curing strategy employing castor oil, modified with isophorone diisocyanate (IPDI) and terminated with 2-hydroxyethyl methacrylate (HEMA) features a photocurable, moisture-reactive chemical structure. The SEM pictures illustrate (a) the dual-cured coating sample, (b,c) photos of produced biobased coatings with 17 wt % of HEMA, and (d,e) photos of produced biobased coatings with 14.5 wt % of HEMA.^112^ Adapted with permission from ref 112. Copyright 2025 Elsevier.

Acrylic systems exemplify the most widely produced, described, and applied radically polymerizable substances for photocuring coatings, due to their availability, fast curing rates, thorough experimental description, and reliable performance. However, itaconic acid represents a biobased, reactive, and attractive alternative and supporting compound for the molecular engineering of the photocurable precursors (see Figure 6).^113^ The recent study employs itaconic acid ester produced by nucleophilic substitution with glycidyl methacrylate (GMA) along with 2-furoic acid-glycidyl methacrylate (FG) in the coating precursors for sustainable protective polymer coatings. Such prepolymer structure exemplifies a nonvolatile, multifunctional photocurable system enhanced by the polymerizable methylidene functional group present in itaconic acid. The authors modified two biobased matrices, 2-furoic and itaconic acid, with GMA, forming a mono- and three-functional methacrylate-itaconate photopolymerizable mixture. Both components play an important role in the formation of the photocurable thermoset coating. While itaconic acid-glycidyl methacrylate (IG) represents a three-functional polymerizable cross-linker enhancing the coating hardness and rigidity, 2-furoic acid-glycidyl methacrylate (FG) comprises a free hydroxyl functional group and a single polymerizable methacrylic acid, providing lower viscosity, better flexibility, and most importantly, sufficient adhesion to the investigated tinplate substrate due to the chemical structure containing a polar, hydrogen-bond-forming functional group. Besides demonstrating the coating protection, the authors also illustrated the recovery and vitrimer-like properties of their proposed reactive system. Due to the presence of free hydroxyl groups in the structure of FG, the thermoset molecular site may be reconstituted at 180 °C and 15 MPa after 1 h of recovery.

**Figure 6:**
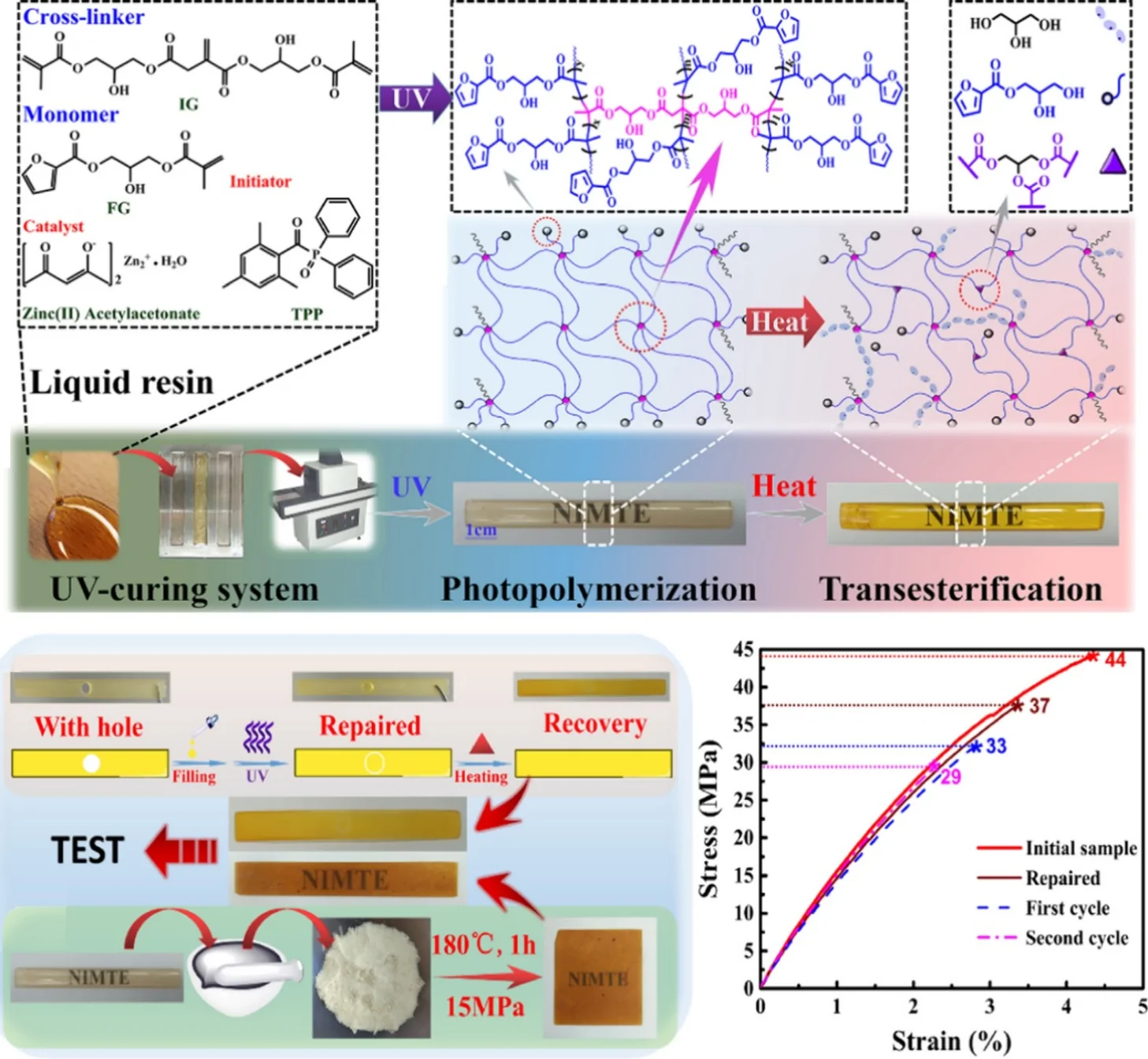
Proposed repairable itaconic acid- and 2-furoic acid-based photocurable systems for polymer thermoset coatings containing free hydroxyl groups capable of repairing the photopolymerized molecular site via a transesterification mechanism catalyzed by the illustrated zinc­(II) acetylacetonate. The vitrimer structure ensures high potential for self-healing coatings through dynamic covalent bonding, which is reprocessable at elevated temperature and pressure.^113^ Adapted with permission from ref 113. Copyright 2019 Elsevier.

The protection and finishes exhibit much wider applicability due to the universal protected surface, ranging from wood adhesives to metal materials requiring anticorrosion protection. The optimal adhesion and tailored mechanical properties are critical for such applications. This perspective provides the following important factors:

- Ideally, the employed polymer coatings for protective purposes and finishes should contain many functional groups (especially polar) to provide exceptional compatibility with various treated surfaces. Selected potential materials, such as nonpolar polymers (PE, PP, PTFE), require a special coating precursor that forms compatible intermolecular attractive bonding.
- Wood coatings exemplify an ideal group of target materials due to their overall compatibility with biobased thermoset polymers. Also, decorative coatings for such substrates often meet the requirements of the furniture-applied color.
- In contrast to electronics and optics, the potential chromophores represent a positive aspect of protective coatings, as they indirectly limit UV-aging of the target adherent.

## 4 Special Applications

Numerous specialized utilities have been reported in the literature, offering biobased polymerizable alternatives for photocurable coatings. Materials from acrylated epoxidized soybean oil precursors used for piezo- and thermoresistive sensing were investigated in the published article. The authors presented photopolymerizable vegetable oil-based precursors that incorporate reduced graphene oxide (rGO) as the functional filler, transforming the polymer molecular sites into a piezoresistive and thermoresistive complex thin-layer system.^114^ The physicochemical properties of the obtained polymeric coatings involved double-bond polymerizable functional group conversion of above 50%, thermal stability up to 300 °C, and the cross-linking density reaching 1.75 mmol·cm^–3^. The proposed piezoresistive and thermoresistive properties, as reported by the authors, serve in various target applications, including automotive, aerospace, and industrial applications to detect structural material damage in real time; robotics and textiles to monitor human functions and robotic motions; and various temperature-sensing applications. As the content of rGO in the polymer coating is investigated, a negative effect of higher additions of the filler is observed. The determined threshold weight content ensures that the thin layers’ operational function requires about 6 wt % of rGO. The Gauge factor (the ratio of the fractional change in electrical resistance to the fractional change in length, or mechanical strain) reached 20 and 26, depending on the deformation, and stabilized at 9 after multiple cycles. The thermoresistive sensibility values reached 0.43 (defined as the relative change in resistance divided by the change in temperature). Eventually, the researchers confirmed the advanced functional purpose of their biobased engineered photocurable coatings employing reduced graphene oxide as an electrically- responsive filler.

Superhydrophobicity and omniphobicity definitely belong among the specialized functional properties of biobased, sustainable photocurable coatings. Balkan et al.^115^ reported an experimental demonstration of the first-mentioned property, proposing photocurable thiol–ene resins incorporating cellulose nanofibrils (CNFs) to replicate the lotus leaf effect as a sustainable alternative to the commonly used fluorinated precursors, which pose significant environmental concerns. The authors incorporated CNFs into a common petroleum-derived photocurable mixture of thiol–ene-forming compounds, trimethylolpropane tris­(3-mercaptopropionate) (TMPTMP), and the monomer trimethylolpropane diallyl ether (TMPDE), triggered by the photoinitiator 2-dimethoxy-2-phenylacetophenone (DMPA). The authors explored numerous physicochemical and functional properties of the prepared coatings containing 0.5–10 vol % CNFs, achieving a measured water contact angle of 155° and exhibiting self-cleaning. Omniphobicity refers to surfaces that repel practically all materials and substances regardless of their surface tension, surface energy, viscosity, or functionality. The authors of the published investigation used the epoxidized soybean oil (ESO) as a reactive, renewable coating precursor mixed with the functional cross-linking agent, tetrakis [(epoxycyclohexyl) ethyl] tetramethylcyclotetrasiloxane (TETC), and an omniphobic agent, polydimethylsiloxane (PDMS), to achieve the omniphobic character of their photocured thin layers (see Figure 7).^116^ The investigated reactive system required a considerably short curing time (20 s), and 0.5 wt % PDMS exhibits remarkable omniphobic properties, which find application in many areas, such as surface chemical shielding, antigraffiti treatment, universal liquid repellency, antifingerprinting, and advanced noctilucent coatings.

**Figure 7:**
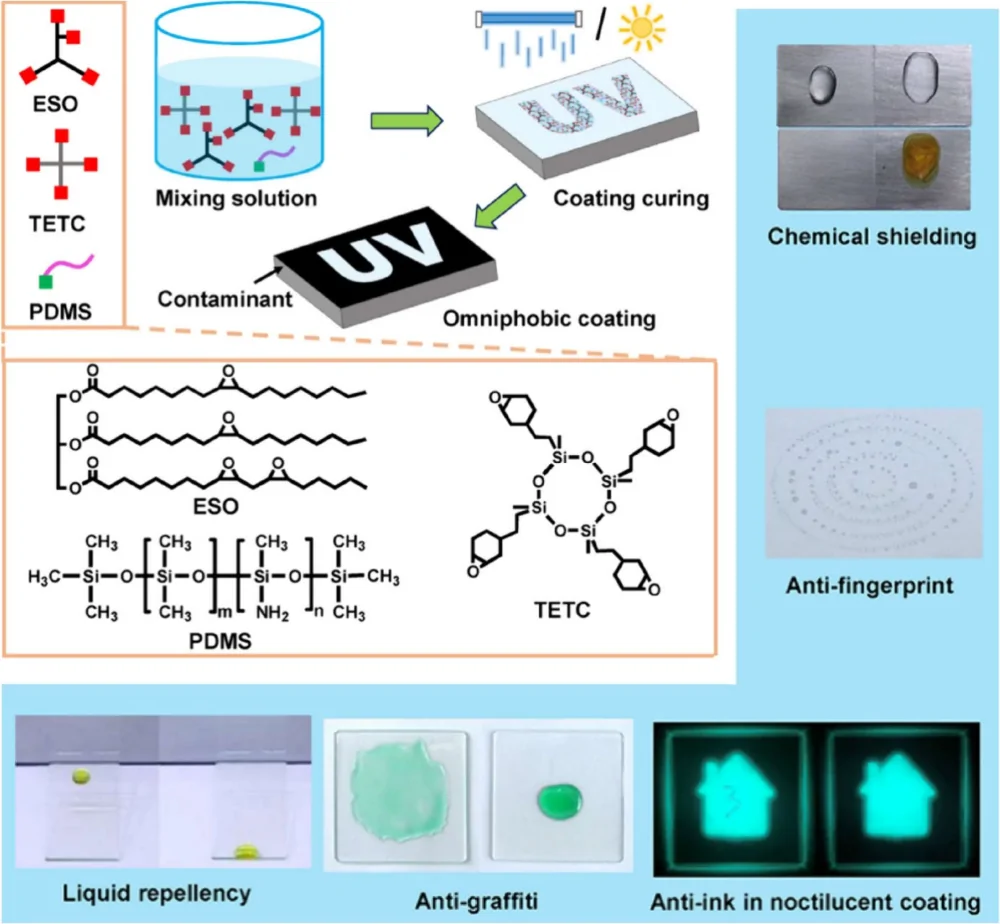
Omniophobic epoxidized soybean oil-based (ESO) photocurable coatings, comprising the functional cross-linker tetrakis­[(epoxycyclohexyl)­ethyl]­tetramethylcyclotetrasiloxane (TETC) and an omniophobic agent, polydimethylsiloxane (PDMS), are presented with their particular applications. The omniphobic coating character ensures the repellency of both water-based polar solvents (hydrophobic) and nonpolar liquid systems (oleophobic).^116^ Adapted with permission from ref 116. Copyright 2025 American Chemical Society.

The available literature also reports specialized photocurable biobased coatings applied in flame-retardant, self-healing complex systems. Chen et al.^117^ developed a solvent- and even photoinitiator-free coating precursor comprising renewable 5-hydroxymethyl furfural (HMF) and thioctic acid (also known as lipoic acid, TA). The catalyst-less coating formation required the synthesis of HMF-TA (a diacetal structure comprising two HMF molecules connected by di­(trimethylol propane) (Di-TMP), terminated by two TA molecules esterified to the acetal complex). Additionally, the DHP-TA compound was employed in the polymer coatings (an ester of TA and diethyl (hydroxymethyl) phosphonate). The DHP-TA component primarily ensured exceptional flame-retardant performance and adhesion. The polymerization mechanism was based on cross-disulfide formation between two separate monomers, HMF-TA and DHP-TA, requiring 365 nm wavelength, 90 mW·cm^–2^ exposure power, 60 °C, and 4 h. The self-healing character was also demonstrated, achieving tensile strength recovery of 92%. The exceptionally long curing times (both for initial UV curing (8 h) and self-healing (4 h)) are due to multiple factors. Primarily, the authors of the investigation demonstrated a photoinitiation-free UV-curing solely forming the 3D molecular structure based on the photoreactive disulfide bonds. The photoinduced cleavage and the dynamic exchange mechanism exemplify the curing process. Compared to photoinitiated polymerization, this strategy requires significantly more process time.^124^ Moreover, the engineered compounds with disulfide bonds were large and complex, which limits their molecular mobility during the UV-curing. Lastly, the authors avoided using a supportive solvent in their polymeric coatings; therefore, the viscosity of their system was excessive, which contributed to the lower curing rates.

The nail coating material segment also demands novel, ideal precursors and application concepts. The research team introduced a multifunctional urethane acrylate hybrid structure designed for the nail coating industry.^118^ Since human nails comprise mainly α-keratin (80–90%), a tough and fibrous protein, containing peptide bonds with hydrogen-bond-forming nitrogen functional groups, several oxygen-involving groups, and generally a strong polar, hydrophilic structure. Due to this chemical composition, nails require specific photocurable precursors that pose minimal health hazards. The authors investigated four different systems: a commercial resin urethane dimethacrylate (UDMA), a biobased acrylated epoxidized soybean oil (AESO), and two synthesized biobased urethane acrylates derived from 1,3-propanediol (PDO–UA) and eugenol (EUG–UA). Commonly, polyurethane materials exhibit exceptional adhesion to many substrates due to their complex, polar, and intermolecular interactions, which form their chemical structure. The worst performance was reached (AESO), as expected, due to the absence of multiple adhesion-enhancing functional groups, poor mechanical performance, and hydrophobic character. PDO–UA outperformed the chosen reference in terms of degree of cure (PDO–UA achieved 96% while UDMA achieved 91%). While the commercial UDMA exhibits primarily rigid, high-glass-transition-temperature behavior, the synthesized PDO–UA and EUG–UA exhibit more flexible, less cross-linked structures. According to the authors, the combination of commercial and newly synthesized systems offers an attractive opportunity to enhance sustainability in the nail-coating industry while allowing tailoring of the material properties of the coatings produced.

UV-absorbing barriers have significant application potential for substrates and adherents that undergo irradiation aging and photodegradation, including wood materials, polymer products, organic pigments and dyes, textiles and fibers, and biocomposites. Among the special application purposes, the photocurable vanillin-based thermosets exhibiting UV-resistant properties were investigated by our research group (see Figure 8).^119^ Vanillin methacrylate was synthesized from vanillin and methacrylic anhydride, employing a biobased, available, and environmentally friendlier catalyst, potassium acetate (KAc), compared to the commonly used 4-dimethylaminopiridine (DMAP). The product yield of 95% was achieved, along with the separation of the formed methacrylic acid (secondary product of the reaction). The separated methacrylic acid was used to synthesize methacrylated vegetable oil from an epoxidized reactive intermediate, using methacrylic acid and triethylamine (TEA) as the nucleophilic substitution catalyst. Different vanillin methacrylate contents were introduced into the reactive systems, namely 0, 10, 20, and 30 wt %. The modified vanillin served as a multifunctional additive, improving the rheological profile, polymerization reactivity, mechanical properties, and adhesion performance, while also providing UV-barrier properties. The absorbed irradiation wavelength reached 306 nm for the methacrylated vegetable oil without a vanillin additive, whereas it extended to 357 nm with 30 wt % vanillin methacrylate in the coating structure. Such an additive is a functional enhancer that provides unique UV-barrier properties.

**Figure 8:**
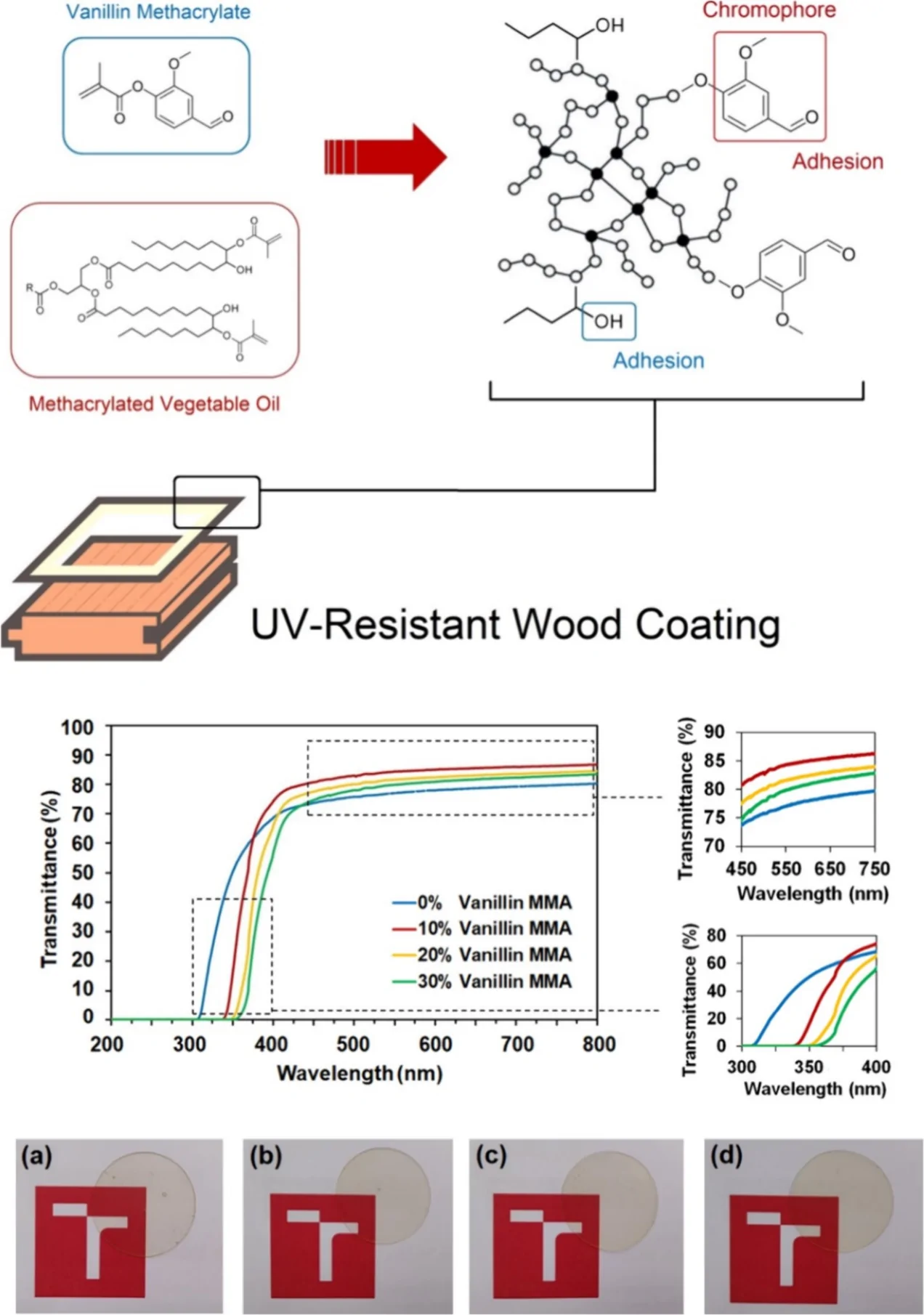
Biobased photocurable coatings comprising methacrylate vegetable oil as the main matrix and vanillin methacrylate as the UV-resistant additive were synthesized by a continuous, waste-free approach, employing environmentally friendlier catalysts and a separated secondary product (methacrylic acid). (a) Polymer coating with 0 wt % of vanillin methacrylate, (b) polymer coating with 10 wt % of vanillin methacrylate, (c) polymer coating with 20 wt % of vanillin methacrylate, (d) polymer coating with 30 wt % of vanillin methacrylate.^119^ Adapted with permission under a Creative Commons CC-BY 4.0 license from ref 119. Copyright 2025 American Chemical Society.

The summarized biobased coatings for specific applications require direct functional character projected through the specific purpose. The essential structural and functional aspects significant for each application are evaluated below:

- **Superhydrophobic Coatings**: functional additives, such as silanes, for decreasing surface energy are irreplaceable in such applications. The overall coating-forming kinetics are a secondary aspect due to the variable production time. The employed epoxides exemplify a slower-curing system.
- **Self-Healing Coatings**: The self-healing mechanism is a critical aspect of such coatings ensured by the presence of disulfide bonds within the thermoset. After permanent deformation, the protective layers are renewed through the UV-triggered strategy.
- **Nail Industry**: The materials for the nail industry should contain a minimal number of health-hazardous compounds. Therefore, the maximal biobased content is required for such applications. Moreover, several toxic and hazardous photoinitiators are used for such applications (e.g., TPO and BAPO), which should be replaced with safer alternatives. Safety here has a higher priority than the coating’s functional performance.
- **Incorporated UV-Barrier**: The combination of curability and UV-absorption requires the presence of specific functional groups (like free hydroxyls demonstrated in the cited investigation) in combination with suitable chromophores (usually a combination of specific functional groups enhanced by the conjugation effect of aromatic rings).

Table 2 summarizes the biobased systems discussed in the mentioned applications. Also, the curing mechanisms are listed in the table.

**Table 2:** Discussed Coating Applications Including the Particular Employed Bio-Based Structure, Concrete Application, and the Curing Mechanism

employed renewable compound	discussed section	application	curing mechanism	ref
palm oil, phytic acid	electronics and optics	triboelectric nanogenerator	radical polymerization	^77^
urea, lauryl acid, cellulose	electronics and optics	electronic heating device and capacitive sensors	radical polymerization	^78^
vanillyl alcohol, eugenol	electronics and optics	integrated circuit packaging	radical polymerization	^79^
epoxidized soybean oil, lignin	protection and finishes	corrosion protection	cationic polymerization	^109^
pentaerythritol, 1,10-decanediol, tetrahydrofurfuryl, camphene	protection and finishes	protective application	radical polymerization	^111^
castor oil, tetrahydrofuran	protection and finishes	wide range of protective application	radical polymerization combined with moisture curing (polyaddition)	^112^
itaconic acid, 2-furoic acid	protection and finishes	protective organic coating	radical polymerization	^113^
epoxidized soybean oil	special applications	piezoresistive and thermoresistive coatings	radical polymerization	^114^
cellulose nanofibrils	special applications	superhydrophobic coating	thiol–ene curing	^115^
epoxidized soybean oil	special applications	omniphobic coating	cationic polymerization	^116^
5-hydroxymethyl furfural, thioctic acid	special applications	flame retardant coatings	photoinduced cleavage and dynamic exchange	^117^
epoxidized woybean oil, 1,3-propanediol, eugenol	special applications	nails industry	radical polymerization	^118^
epoxidized soybean oil, vanillin	special applications	UV-barrier coating	radical polymerization	^119^

## 5 Conclusion and Future Outlook

This work discusses the utility potential of reactive, photocurable compounds and systems engineered from renewable sources, designed for coating applications across the commercial sphere and materials industry. As reported, numerous experimental investigations were conducted to verify the suitability of various natural templates for chemical modification and to demonstrate their potential for thin-layer applications. Among the investigated compounds and systems, triacylglycerides, natural phenolic compounds, bioderived carboxylic acids, and renewable polyols played the most significant role due to their potential for esterification (carboxylic acid, modified triacylglycerides, hydroxy derivatives), epoxidization (unsaturated triacylglycerides and unsaturated phenols), polyaddition (hydroxyl-involving systems and bioderived isocyanates), and thiol–ene reaction (sulfur-containing derivatives and unsaturated compounds with a methylidene functional group). In most cases, the functionalized biobased reactive precursors played a role as a continuum, a main photocurable matrix, and an assisting system. Specified additives and functional systems mostly ensure the application potential, such as dispersed silver particles, nano- and microparticle cellulose, and polydimethylsiloxane (PDMS).

Based on the published investigations, several natural templates for photocurable coatings exhibit specific functional properties, thereby offering added value for the electrical products to which they are applied. Incorporated phytic acid ensured unique electrical properties for triboelectric nanogenerators (TENGs); the combination of urea-bonded oil-based “soft” segments and cellulose-derived “hard” segments ensured optimal functional properties in capacitive sensors; and eugenol–vanillin epoxy-thiol–ene cross-linked photocured thermoset formed an ideal material for electronic packaging coatings. Optimal protective shielding coatings derived from renewable sources were successfully applied to a range of substrates, including wood, glass, metals, and polymers. The abrasion-protective, anticorrosive, antibacterial, and designer properties were achieved while maintaining considerable biobased structural content in the employed systems. Finally, special advanced and functional properties were described, including a dual photomoisture-curing strategy, piezo- and thermoresistive sensing, omniphobicity, and an integrated UV-barrier.

This perspective offers several observable advantages of the employed biobased materials and utilized renewable compounds. In particular, the substituted fossil-based polymers with biobased alternatives exhibit the following specifics:

- Biobased polymeric coating precursors contain a significant amount of renewable carbon, which contributes to the sustainable strategy currently emphasized across material industries and most applications.
- Suitable highly functional reactive coating precursors with medium- or long-chain carbon backbones, ensuring low volatility and a chemical composition (functional groups) that decrease environmental impact and exemplify a potentially safer system from a health hazard standpoint.
- The optimal combination of naturally more rigid substances after modification (aromatic compounds and specific cycloaliphatic compounds) with long-chain derivatives that confer greater flexibility (modified lauryl acid or triacylglycerides) ensures biobased polymer coatings with a unique material character for numerous applications.
- Several compounds from renewable sources contain multiple prospective functional groups (free hydroxyl groups, carboxyl groups, disulfide bonds, ester bonds, and others) that could serve as a platform for vitrimer systems. Vitrimers and other triggered complex systems could have a circular character, minimizing the need for direct disposal of the material.
- Instead of combining multiple additives in the final product to ensure an optimal rheological profile of the applied polymer precursor coating, sufficient mechanical properties of the formed layer, and the target function, selected multifunctional compounds from renewable sources ensure all mentioned requirements in a single molecule (vanillin methacrylate provides the optimizing of flow properties, improves the rigidity of the coating, and ensure the UV-absorption due to its unique chemical structure).

The future application potential of many reported, introduced, and described compounds and photocurable multicomponent systems is targeted for commercial demand. This work demonstrates the suitability of renewable sources not only for substituting for fossil-based templates, which serve only as a working continuum for further functional components, but also for various components that exhibit essential, unique, and cutting-edge functions. The main challenges for the efficient employment of the biobased precursors in real material applications are discussed below:

(1) The photopolymerization activity of biobased systems is usually lower than that of fossil-based commercial systems. The combination of highly reactive functional groups and a renewable carbon backbone can ensure the optimal balance of multiple required properties. Ideally, a fully biobased structure incorporating renewable polymerizable groups could be engineered, but the photopolymerization rate of such a compound would be challenging. Additional low-molecular additives that decrease viscosity and increase molecular mobility may improve performance.
(2) Renewable compounds with sufficient purity are often more expensive than the currently employed precursors. The valorization of waste entering sources may decrease overall costs; however, the quality of the entering compounds should be monitored.
(3) Several biobased curable precursors exhibit specific colors due to their complex structures, which contain multiple chromophores. While some applications, such as protective coatings, finishes, and UV-barriers, benefit from this factor, electronic and optical applications often require a highly transparent system with limited visible-light absorption. When highly transparent coatings are required, several modifications of the biobased system may be demanded.
